# Supplementary material for: Comprehensive Annotation and Functional Exploration of MicroRNAs in Lettuce
Source: Front Plant Sci. 2021 Dec 24;12:781836. doi: 10.3389/fpls.2021.781836 (PMC8739914; doi:10.3389/fpls.2021.781836)
Supplement: Supplementary file 2 [file Data_Sheet_8.ZIP › GSM2230747.plot/Lsa-miRN1699b_Lsat_1_v5_gn_5_13561.1_1234_TPlot.pdf]

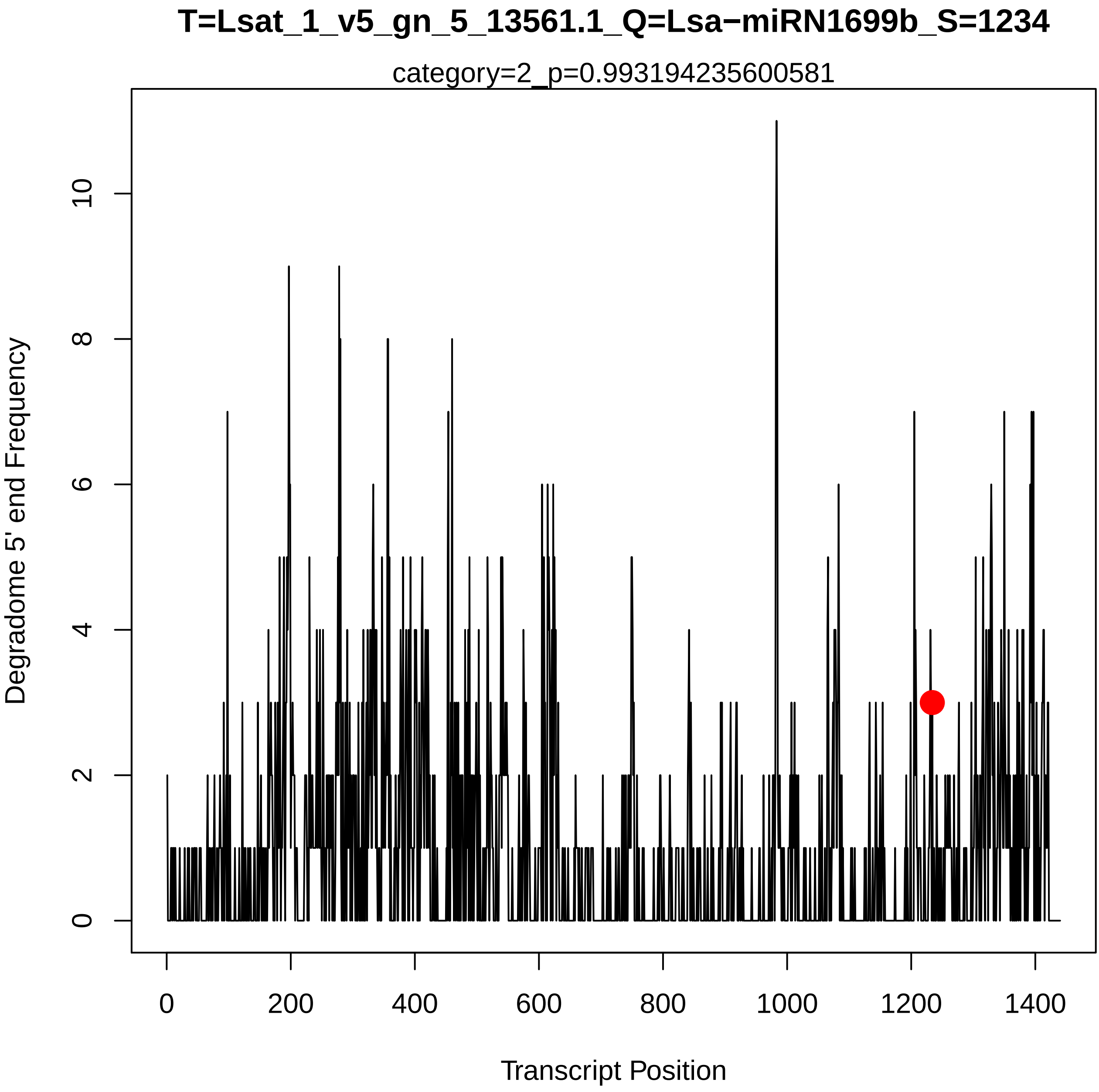
T=Lsat_1_v5_gn_5_13561.1_Q=Lsa−miRN1699b_S=1234
category=2_p=0.993194235600581
Degradome 5' end Frequency
0
2
4
6
8
10
Transcript Position
0
200
400
600
800
1000
1200
1400
